# New Insights and Challenges Associated With IgA Vasculitis and IgA Vasculitis With Nephritis—Is It Time to Change the Paradigm of the Most Common Systemic Vasculitis in Childhood?

**DOI:** 10.3389/fped.2022.853724

**Published:** 2022-03-15

**Authors:** Marija Jelusic, Mario Sestan, Teresa Giani, Rolando Cimaz

**Affiliations:** ^1^Department of Paediatrics, University of Zagreb School of Medicine, University Hospital Centre Zagreb, Zagreb, Croatia; ^2^Department of Clincial Sciences and Community Health, University of Milan, Milan, Italy; ^3^ASST Pini-CTO, Milan, Italy

**Keywords:** IgA vasculitis, IgA vasculitis nephritis, epidemiology, pathogenesis, clinical presentations, diagnostics, disease activity, treatment

## Abstract

What are the challenges ahead and how have we responded so far when it comes to the non-granulomatous systemic vasculitis, characterized mainly by deposits of IgA immune complexes in the endothelium of small blood vessels—IgA vasculitis (IgAV)? That is the question to which we tried to answer. We summarized existing knowledge about epidemiology, pathogenesis, genetics, diagnostic tests and therapy in this somewhat neglected entity in pediatric rheumatology. Since etiopathogenesis of IgA vasculitis is complex, with factors other than galactose-deficient IgA_1_-containing immune complexes also being important, and may involve numerous interactions between environmental and genetic factors, genomics alone cannot explain the entirety of the risk for the disease. The incidence of IgAV and nephritis varies worldwide and may be a consequence of overlapping genetic and environmental factors. In addition to the role of the HLA class II genes, some studies have pointed to the importance of non-HLA genes, and modern geostatistical research has also indicated a geospatial risk distribution, which may suggest the strong influence of different environmental factors such as climate, pathogen load, and dietary factors. The application of modern geostatistical methods until recently was completely unknown in the study of this disease, but thanks to the latest results it has been shown that they can help us a lot in understanding epidemiology and serve as a guide in generating new hypotheses considering possible environmental risk factors and identification of potential genetic or epigenetic diversity. There is increasing evidence that an integrative approach should be included in the understanding of IgA vasculitis, in terms of the integration of genomics, proteomics, transcriptomics, and epigenetics. This approach could result in the discovery of new pathways important for finding biomarkers that could stratify patients according to the risk of complications, without an invasive kidney biopsy which is still the gold standard to confirm a diagnosis of nephritis, even if biopsy findings interpretation is not uniform in clinical practice. Ultimately, this will allow the development of new therapeutic approaches, especially important in the treatment of nephritis, for which there is still no standardized treatment.

## Introduction

IgA vasculitis (IgAV) is a non-granulomatous systemic vasculitis, histologically characterized by infiltration of the walls of the blood vessels, mainly arterioles, capillaries and venules, by neutrophils with deposits of immune complexes containing predominantly IgA (1–4). The endothelium of small blood vessels in the skin, synovial membrane, gut, and kidneys is usually involved (2).

Even though IgAV is the most common form of vasculitis in childhood (5), it is somewhat neglected in pediatric rheumatology, as it is mostly perceived as a self-limited disease lasting up to 4 weeks, with care for these patients scattered between nephrologists, rheumatologists, dermatologists, and gastroenterologists (6).

It is important to keep in mind that despite the favorable prognosis for most pediatric patients, various acute and chronic complications are possible (7, 8). Among the acute complications of IgAV, the most frequent are those related to the gastrointestinal system, including bleeding, intussusception, and bowel perforation as the most serious ones (9). The most important chronic complication and the main cause of morbidity and mortality among children suffering from IgAV is the renal aspect of the disease (IgAV nephritis, IgAVN), which therefore represents the main prognostic factor (8). IgAVN occurs in 20–60% of children suffering from IgAV, and among them chronic renal failure has been reported in 1–15% (10–14).

We will try to show how new insights has been gained regarding this disease, including epidemiology, pathogenesis, genetics, diagnostic trials, and therapy, as well as to point out the fact that many questions and dilemmas concerning IgAV remain unanswered.

## New Insights in the Epidemiology of IgAV

**Figure 1:**
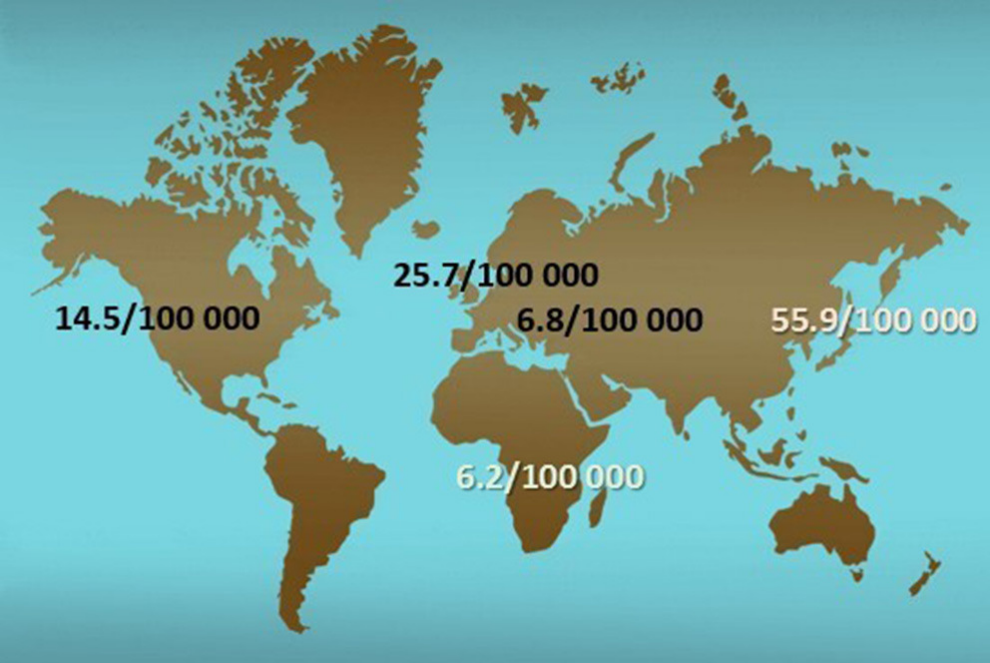
Distribution of incidence of IgAV around the world.

It is known that the incidence of IgAV varies worldwide ranging from 3 to 55.9 cases per 100,000 children (Figure 1), while the prevalence varies between 6.1 and 20.4 per 100,000 children (2, 15–17). The fact that IgAV is not equally present in different parts of the world is also well-known, since it has the highest occurrence in East Asians, intermediate in Europeans, and the lowest in individuals of African ancestry (2, 15). Nevertheless, only recently a study on spatial variability of the incidence of IgAV and IgAVN using modern geostatistical methods has been performed (18). This research is important for several reasons. First, there is a lack of application of geostatistical methods in the field of pediatric rheumatology at all. Most of the previous work explores potential risk factors using observational studies with classical statistical techniques resulting in reduction of information which may be used to deepen the knowledge of potential risk factors involved in the pathomechanism of disease, in this case IgAV. In other words, the geospatial analysis may provide useful information of genetic, socio-economic, and environmental risk factors while simultaneously taking into account their spatial diversity, which can be applied not only in the case of IgAV and IgAVN but also in many other diseases. Second, it was demonstrated that both IgAV and IgAVN may not be randomly distributed in space, but clustered, similar to some other non-communicable diseases, such as inflammatory polyarthritis, heart diseases, and diabetes (18–21).

The IgAV and IgAVN hotspots clusters appear where genetic and environmental factors overlap substantially. It can be speculated whether genetic or environmental factors are dominant in the example of IgAV and IgAVN clustering in Croatia (18, 22). Since IgAVN showed linear clustering in the eastern part of Croatia, which follows the course of the Drava and Danube rivers, in the vicinity of areas of Balkan endemic nephropathy, known to occur primarily due to environmental factors (aristolochic acid) (23), the question arises as to whether the same is true for IgAVN. If this proves correct, it would be contrary to geospatial distribution of IgA nephropathy that is predominantly associated with genetic factors—different variants of innate immunity genes as well as of genes important for defense against parasitic infections (24). That would be another important difference between IgAVN and IgA nephropathy, for which it is still debated whether they are different diseases or just two variants of the same disease (25).

## New Insights in the Pathogenesis of IgAV

The complexity of the etiopathogenesis of IgAV is reflected in the interaction of genetic and environmental factors, with special emphasis on infections (16, 26). The genetic background is indisputable; this is supported by the fact that the incidence and geospatial distribution of IgAV and IgAVN differ around the world and between the different ethnicities (2), that the incidence of IgAV sometimes has a tendency for familial aggregation (27–29), and, finally, that genome-wide association studies (GWAS) point to the significance of common gene variants in the pathogenesis of this disorder (30, 31).

The results of the GWAS to date classify IgAV as a prototype of a disease related to human leucocyte antigen (HLA) class II loci. A first GWAS pointed to the significance of the polymorphisms in the *HLA-DQA1* and *DQB1* intergenic zone and at the HLA-DRB1^*^11 and DRB1^*^13 loci (30), while a more recent one showed that haplotype DQA1^*^01:01/DQB1^*^05:01/DRB1^*^01:01 was associated with susceptibility to IgAV but not with other autoimmune diseases (31). GWAS of IgAV have not detected potential susceptibility loci to IgAV outside HLA class II genes, but since there are no large GWAS for pediatric IgAV and IgAVN, and existing studies are underpowered to detect smaller allelic effects outside of the HLA region, it is possible that variants in various non-HLA genes associated with immune and inflammatory response escaped statistical detection and these are variants that previous studies have shown to be implicated in the etiopathogenesis of IgAV (32). The most important non-HLA genes linked with IgAV susceptibility include cytokines genes (ILRN^*^2, IL18, and *TGFB1*), chemokines genes (*MCP1*), adhesion molecules genes (*SELP*), renin-angiotensin system (*RAS*) genes (*Agt, ACE*), and others (*C1GALT1, NOS2A, eNOS, PON1*, and *MEFV*) (32). For example, genetic variants located at the interleukin (*IL*) 18 locus could be associated with a higher risk of developing IgAVN (33), while carriage of interleukin-1 receptor antagonist polymorphism 2 (ILRN^*^2) may be related to severe renal involvement and renal sequelae in patients with IgAV (34). Even though the results of these studies deliver more insight into the various molecular pathways, they are not sufficiently large, and the potential association with IgAV is not as strong as it is the case with HLA genes.

These genetic variants may be responsible for regional and ethnic differences observed in IgAV. Thus, for example, the pathogenic variants in the Mediterranean fever (*MEFV*) gene might predispose to IgAV in patients with familial Mediterranean fever (FMF) and may be associated with different clinical presentation of IgAV in countries where FMF is common (35, 36). In these patients, there might be an increased risk of gastrointestinal complications and IgA deposits on biopsy specimens might be less frequently found, so the diagnosis of IgAV should not be excluded based on absent IgA deposits on skin biopsy in case of clinical presentation suggestive of IgAV (37).

It was shown that epigenetic changes, that regulate gene activity and expression, are involved in pathogenesis of IgAV (38). Luo et al. demonstrated increased global histone H3 acetylation and H3K4 methylation levels in the peripheral blood mononuclear cells of patients with IgAVN compared to healthy controls and patients with IgAV without renal involvement. Furthermore, the authors showed positive correlation of H3 acetylation and H3K4 methylation levels with disease severity. They hypothesized that abnormal levels of histone modifying enzymes can lead to changes in chromatin structure, resulting in increased gene transcription, such as the IL-4 promoter. Another important finding in this study was that in patients with IgAV, the balance between type 1 helper cells (Th1) and type 2 helper cells (Th2) cytokines is disturbed by increasing the level of Th2 specific cytokines (IL-4, IL-6, and IL-13) and decreasing the level of Th1 specific cytokines (IL-2 and IFN-γ) (38).

Recently, a gene which encodes a histone demethylase involved in the epigenetic control of gene expression (*KDM4C*) has been implicated in the genetic predisposition of IgAV, highlighting the relevance of the epigenetic mechanisms in the development of this disease (39).

**Figure 2:**
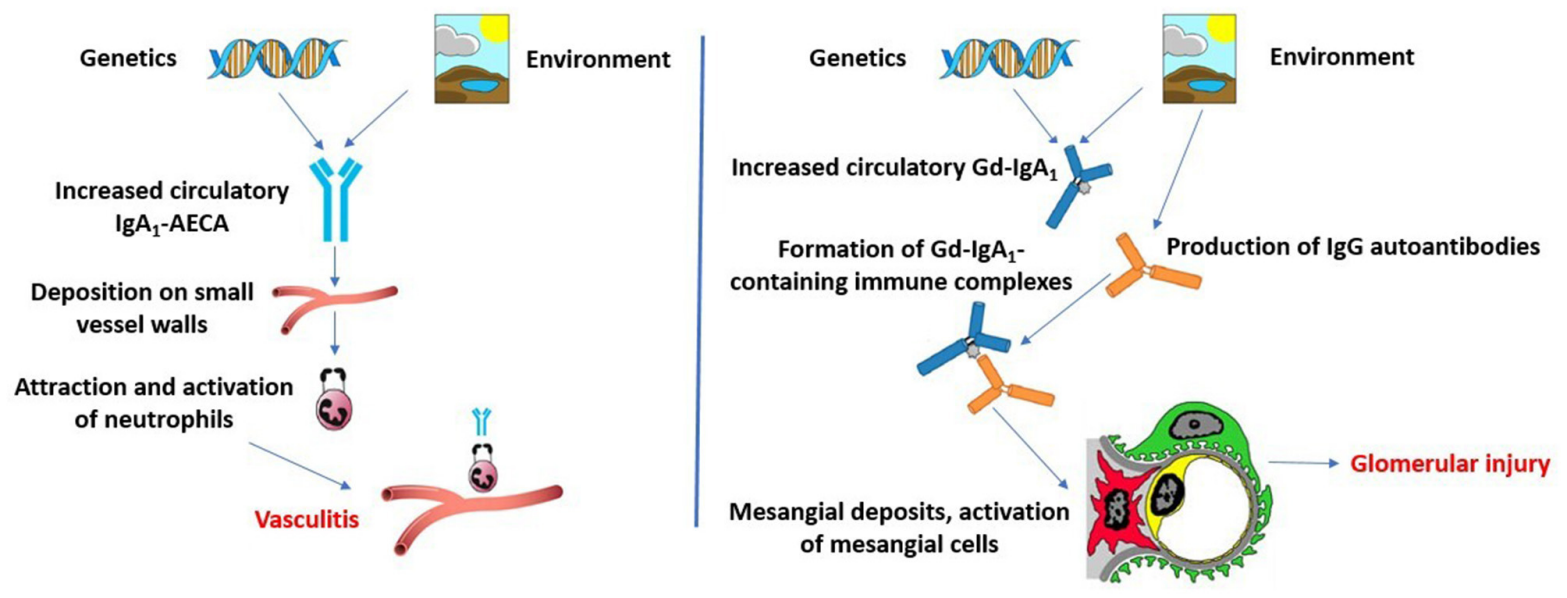
Two multi-hit pathogenesis models for IgAV and IgAVN. Modified according to Heineke et al. (40) and Hastings et al. (41).

Multi-hit pathogenesis models for IgAV and IgAVN have been described lately (40, 41) (Figure 2). It seems that the aberrantly glycosylated IgA1 plays a key role in the pathogenesis of IgAV, especially in patients who develop IgAVN. IgA_1_ from most patients with IgAV lack galactose residues [galactose deficient IgA_1_ (Gd-IgA_1_)] (42). It is hypothesized that in the Golgi apparatus of IgA_1_-producing immune cells aberrant glycosylation occurs due to decreased galactosyltransferase activity and that genetic predisposition and/or mucosal infection and concomitant IL-6 production cause aberrant glycosylation by altering the glycosylation machinery (43). Two potential genetic loci have been identified in a GWAS in adult patients with IgA nephropathy and increased serum levels of Gd-IgA_1_ (44). These loci, *C1GALT1* and *C1GALT1C1*, are inherited in an autosomal dominant manner and may be responsible for aberrant glycosylation in IgAV, and not only in patients with IgA nephropathy. IgA and IgG antibodies may recognize Gd-IgA_1_ as an autoantigen, which leads to the formation of polymeric immune complexes (Gd-IgA_1_-IgA, Gd-IgA_1_-IgG, and Gd-IgA_1_-sCD89, where sCD89 is soluble IgA Fc alpha receptor). It is possible that these circulating immune complexes accumulate in the blood, resulting in their deposition on the endothelium of small blood vessels in the skin, gut, and kidneys. It was shown that serum levels of Gd-IgA_1_ are higher in IgAVN patients compared to IgAV patients without nephritis (45). Some of these Gd-IgA_1_-IgG complexes may deposit in the kidneys, resulting in mesangial cell activation, release of inflammatory mediators, and glomerular injury in patients who develop IgAVN (41).

However, some authors proposed a second multi-hit hypothesis to explain the systemic symptoms of IgAV and IgAVN (40). This model is based on assumption that infection with microorganisms that have similar antigenic structures as components of human vessel walls could lead to the production of cross-reactive anti-endothelial cell antibodies (IgA_1_-AECA) under specific genetic influences. These antibodies may further induce the production of interleukin-8, which is a potent chemoattractant for neutrophils. After activation, neutrophils may cause damage of vascular endothelial cells.

In the context of the new insights, and related to the coronavirus disease 2019 (COVID-19) pandemic, it should be noted that there are several case reports describing patients with IgAV following COVID-19, and some of them were children (46–48). It is not yet known how the severe acute respiratory syndrome coronavirus 2 (SARS-CoV-2) virus is involved in the pathogenesis of IgAV, whether it is a classical infectious trigger such as the previously described bacteria and viruses or because of its ability to elicit a cytokine response it may be directly involved in the pathogenesis of the disease.

None of the proposed models of IgAV pathogenesis described can explain the fact that in <10% of IgAV patients Gd-IgA_1_ in serum or in biopsy specimens is not elevated, nor can they explain the observation that the disease occurs only in some people with IgA_1_ glycosylation defect, while in others with elevated Gd-IgA_1_ the disease does not occur (49, 50).

## New Insights in the Clinical Presentations of IgAV

**Figure 3:**
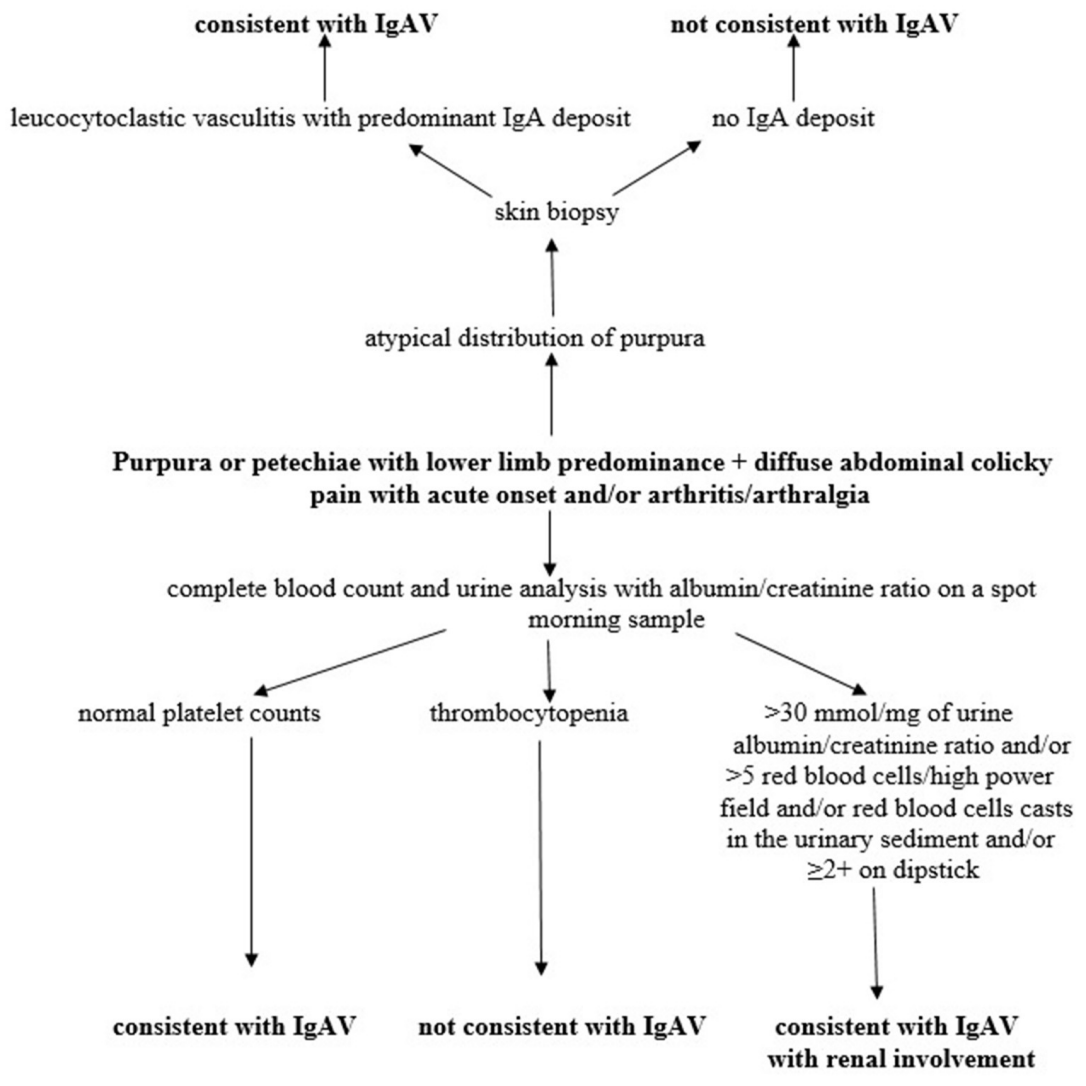
Diagnostic algorithm of IgAV. Modified according to Ozen et al. (2).

The most common and characteristic signs of the disease are skin manifestations in the form of non-thrombocytopenic purpura or petechiae with lower limb predominance. They are present in all patients and are mandatory classification criterion according to the European League Against Rheumatism (EULAR), Pediatric Rheumatology International Trials Organization (PRINTO) and Pediatric Rheumatology European Society (PRES) classification criteria endorsed in Ankara in 2008 (2). A possible diagnostic algorithm of the disease is shown in Figure 3.

Nonetheless, atypical distributions are also possible, affecting the head and neck area, involving the upper extremities more than lower extremities, sparing of the lower extremities, or with diffusely distributed lesions (51). Furthermore, hemorrhagic bullae, ulcerations and necrotic lesions can be seen in the most severe cases. Recently, several papers have been published on severe skin changes in patients with IgAV, questioning whether these changes are associated with more severe disease, persistent sequelae, and discussing how to treat patients with such manifestations (52–54). The results of these studies are not uniform, and according to some authors the presence of ulcerations and necroses, persistent purpura (≥1 month) and older age were significant predictors of IgAVN. Also, with increasing severity and duration of cutaneous manifestations in IgAV the risk of developing IgAVN may increase, with a greater likelihood to need aggressive treatment (50). However, others did not notice such associations (52, 53). All the studies conducted so far have in common that they included a very small number of IgAV patients with the most severe cutaneous manifestations, so it is necessary to wait for further results to reach conclusions that will include a larger number of patients.

The second most common feature is represented by musculoskeletal manifestations, in that up to 70–90% of patients with IgAV will have arthralgia or arthritis (51). According to a Korean nationwide population-based study, younger children are more at risk of developing arthritis, and children younger than 7 years of age had frequent joint symptoms (17). In children with IgAV arthritis is non-deforming and heals without chronic damage within a few weeks (51). It is interesting to note that in some studies it was observed that joint involvement and subcutaneous edema in the extremities were less frequent in patients with severe gastrointestinal involvement, thus arthralgia could be a negative predictive factor for severe gastrointestinal involvement in patients with IgAV (55).

More than 50% of children with IgAV may develop gastrointestinal manifestations, and in about 10–20% of patients with gastrointestinal involvement serious complications such as intussusception, bowel perforation, and massive bleeding may occur (9). There are conflicting results from literature regarding the possible association of gastrointestinal symptoms in IgAV and IgAVN. A meta-analysis from 2016 showed that gastrointestinal symptoms were strongly related to renal involvement (56), and several other studies have shown similar results (57–59). Patients in whom IgAV has started with gastrointestinal symptoms and older children with severe gastrointestinal symptoms (severe abdominal pain, intussusception, hematochezia, and/or massive gastrointestinal bleeding) may be a high-risk group for developing IgAVN (59). However, other studies have not confirmed this association (60–62).

Finding associations of individual clinical elements with the prognosis of IgAV and renal involvement would be very important since it would help to identify high risk group of patients that should be follow-up closely and at shorter intervals to detect renal involvement.

## New Insights in the Diagnostics of IgAV, With Special Emphasis on the Role of Biomarkers and Kidney Biopsy

Renal involvement in IgAV ranges from urinary abnormalities (including hematuria or/and proteinuria) through nephritic and nephrotic syndrome to chronic renal failure. IgAVN is typically mild and most commonly manifest only with pathological urine findings. Chronic renal failure has been reported in 1–15% of children with IgAVN, and in the vast majority of cases it is diagnosed within 6 months of disease onset (11–14).

Current problems related to the diagnosis of renal disease in IgAV can be divided into two groups. On one hand, kidney biopsy is still the gold standard for the diagnosis of IgAVN, but the big problem is the uneven interpretation of the results since several histological classifications are used in the analysis of renal biopsy findings in IgAVN, but it remains unknown which one has the strongest association with the severity and outcome (6). Another problem is that there are currently no biomarkers in routine clinical use for IgAV and IgAVN which can stratify patients with respect to the risk of developing kidney disease progression and contribute to the earlier diagnosis of renal involvement (41).

The most commonly used histological classification is that of the International Study of Kidney Disease in Children (ISKDC) (63, 64). The advantage of this classification is that it is relatively simple and widespread in use, so it is known around the world (6). The most important limitation is that it is grounded mostly on the state of glomeruli, therefore only reflecting active inflammation and neglecting vascular and tubulointerstitial changes. The Oxford classification is increasingly used, and was revised in 2017 (65). Crescents, the lesions on renal biopsy that have long been considered the most important outcome indicators of IgAVN, were not included in the first version of the classification. Although this histological classification is more complex and some studies have shown its potential for use in IgAVN, caution is needed. Indeed, the working group of Oxford classification does not recommend its use in IgAVN since cases of patients with this condition were not included in the validation cohort, and recent study suggests that the Oxford classification could not be fully validated in IgAVN (65, 66). A finnish group published the modified semi-quantitative classification in 2017 (67). It is the most complicated, but the first to take into account the chronicity components. Although the first results are promising, a study of a longer number of patients is needed to properly validate the classification.

Since renal biopsy is an invasive procedure, and it is still unclear what is the prognostic value of individual histological elements, there is a need for less invasive procedures in diagnostics. Measurement of biomarkers in urine has many advantages: the sample is relatively easy to collect, without using invasive procedures; urine reflects changes in renal parenchyma, unlike blood that is in contact with a number of organs and organ systems. In addition, the number of different core proteins in the urine is lower than in blood (68, 69). Despite the many potential biomarkers that are emerging, the most consistent finding in patients with IgAV remains an increased serum level of Gd-IgA_1_ (70). Among urinary biomarkers, IgA and IgM performed best in the study conducted by Pillebout et al. (45). Recent systematic review of urine biomarkers in children with IgAVN showed that the most promising urinary biomarkers in predicting nephritis were kidney injury molecule-1 (KIM-1), monocyte chemotactic protein-1 (MCP-1), N-acetyl-β-glucosaminidase (NAG), and angiotensinogen (AGT) (71). However, none of them proved yet to be an established marker of disease. Further studies are needed to verify whether preclinical markers are superior to the currently usd ones (24-h urinary protein values, urinary protein:creatinine ratio and urinary albumin concentration).

The application of metabolomics has proven to be a promising approach (72). Metabolome is a product of proteome and is considered to be closer to the phenotype in comparison with genome, and proteome. Studies regarding metabolomics in IgAV are scarse. Demir et al. found that DHAP (18:0), prostaglandin D2/I2, porphobilinogen, 5-methyltetrahydrofolic acid, and N-Acetyl-4-O-acetylneuraminic acid/N-Acetyl-7-O-acetylneuraminic acid may serve as biomarkers for predicting kidney disease but studies with larger number of IgAV patients are necessary for validation of these findings (72).

## New Insights in the Assessment of Disease Activity and Damage in IgAV

Data regarding vasculitis activity and damage assessment in children with IgAV are limited. Since these are key components of outcome measures in patients with vasculitis for both clinical trials and for observing individual patient disease course, it is important to validate the available assessment tools adapted for the pediatric population (73). For determining disease activity and degree of kidney damage in patients with IgAV/IgAVN two clinical questionares can be used: the Pediatric Vasculitis Activity Score (PVAS) and Pediatric Vasculitis Damage Index (PVDI), respectively (73). PVAS includes 64 clinical features (symptoms or signs of disease) that are divided into nine organ systems; the assessment of new or worsening items in the last 4 weeks, but not for more than 3 months, is recorded. The total number of points represents the activity of the disease and ranges from 0 to 63 points. PVDI includes 72 clinical variables divided into nine organ systems, as well as an “other” section. The duration of symptoms or signs lasting at least 3 months, occurring at any time since the disease onset, is defined as damage.

## New Insights in the Treatment of IgAV

During the self-limited nature of the disease, in the vast majority of children with IgAV specific treatment is not required. Regarding patients with severe skin manifestations, due to the lack of studies with large number of participants the optimal way of treatment is not known, although most are treated with systemic glucocorticoids, sometimes in combination with dapsone or azathioprine (52, 53). Musculoskeletal manifestations are usually treated with rest and analgesia, while other treatment options are rarely necessary. A different situation occurs in children with severe gastrointestinal manifestations, renal involvement or those with other complications such as neurological, lung or multiple organ involvement. Recently, the European initiative SHARE (Single Hub and Access point for pediatric Rheumatology in Europe) developed consensus-based recommendations for diagnosis and treatment of IgAV (74). It is important to emphasize that these recommendations are not intended for pediatric rheumatologists and nephrologists, but for general pediatricians and physicians who have little or no experience with severe IgAV and IgAVN patients.

In children with severe abdominal pain or gastrointestinal hemorrhage, glucocorticoids should be considered: oral or pulsed glucocorticoids if oral route is not tolerated or they have failed to respond. Mycophenolate mofetil, cyclophosphamide, intravenous immunoglobulin, rituximab, methotrexate, colchicine, and hydroxychloroquine may be considered as second-line treatments (51). Other supportive treatment, such as, nasogastric decompression, parenteral nutrition, and antibiotics may be required.

**Figure 4:**
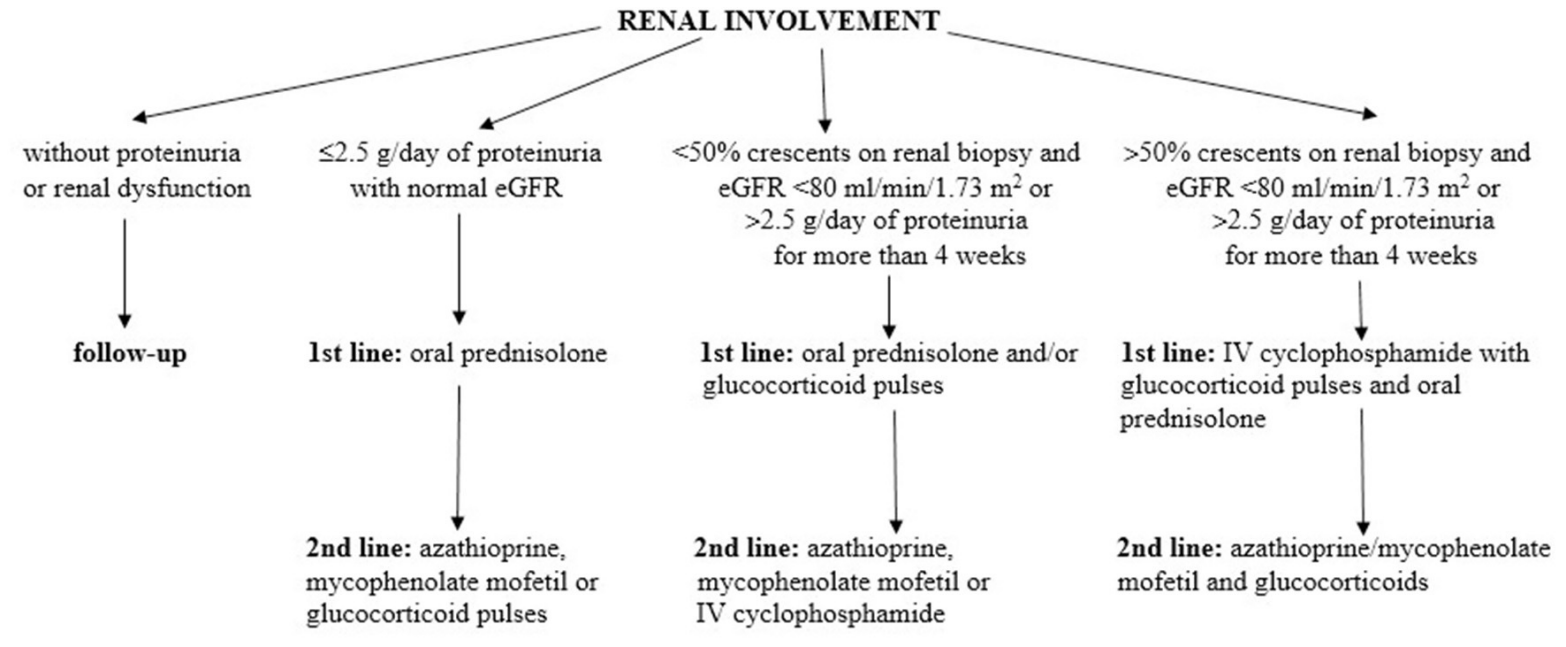
The SHARE recommendations for IgAVN treatment. Modified according to Ozen et al. (74).

According to the SHARE management algorithm, IgAVN is divided into three categories, taking into account proteinuria, estimated glomerular filtration rate and percentage of crescents on renal biopsy (74). Children without proteinuria or renal dysfunction usually do not need any specific therapeutic intervention. In patients with mild forms of IgAVN, defined as ≤ 2.5 g/day of proteinuria in 24 h urine collection with normal estimated glomerular filtration rate, first-line therapy consists of oral glucocorticoids, and in the case of persistence of proteinuria, second-line drugs may be used (e.g., azathioprine, mycophenolate mofetil, or glucocorticoid pulses). Glucocorticoids, usually parenterally and in pulsed doses, are the first choice in the treatment of moderate IgAVN, defined as <50% crescents on renal biopsy and impaired estimated glomerular filtration rate (<80 ml/min/1.73 m^2^) or severe persistent proteinuria (>2.5 g/day of proteinuria in 24 h urine collection for more than 4 weeks). In the absence of response, second-line drugs are added: azathioprine, mycophenolate mofetil, or cyclophosphamide parenterally. Treatment of the most severe forms of IgAVN consists of two phases: the first one is induction using pulsed doses of glucocorticoids in combination with intravenous cyclophosphamide pulses, and the second phase is maintenance therapy with lower doses of glucocorticoids in combination with immunomodulators: azathioprine or mycophenolate mofetil. To prevent or limit secondary glomerular damage in patients with IgAVN who have persistent proteinuria (lasting more than 3 months), angiotensin converting enzyme inhibitors or angiotensin receptor blockers are recommended. The SHARE recommendations for IgAVN treatment are summarized in Figure 4.

For unresponsive cases there is an option of plasma exchange that showed efficacy in one study while there is not enough evidence regarding the use of rituximab (although it has been described in case reports and case series) or intravenous immunoglobulins (51, 75).

In addition to the SHARE recommendations, there is also the Kidney Disease: Improving Global Outcomes (KDIGO) practice guideline on glomerulonephritis, which in one chapter provide recommendations for the treatment of IgAVN in children and adults (76). Angiotensin converting enzyme inhibitors or angiotensin receptor blockers are suggested for children with persistent proteinuria 0.5–1 g/day per 1.73 m^2^, while for those with proteinuria >1 g/day per 1.73 m^2^, after a trial of angiotensin converting enzyme inhibitors or angiotensin receptor blockers, a 6-month course of glucocorticoid therapy should be considered. Children with crescentic IgAVN with nephrotic syndrome and/or deteriorating kidney function should be treated with glucocorticoids and cyclophosphamide according to KDIGO practice guideline.

The biggest problem in treating children with IgAV is the lack of high-level evidence and randomized controlled trials (74).

When it comes to new therapeutic options, there is room for improvement. Since the most important chronic complication of the disease is IgAVN, and according to some hypotheses the pathogenesis of IgAVN could be closely related to the pathogenesis of IgA nephropathy (40, 41, 43), new targeted therapies being investigated in IgA nephropathy could soon be extended to IgAVN. Examples of such therapeutic options include budesonide, which can target Peyer's patches in the ileum where the production of Gd-IgA1 is thought to originate (77); bortezomib, a proteasome inhibitor which is a plasma cell depleting agent (it affects production of IgG autoantibodies) (78); complement inhibitors such as APL-2, CCX168, LNP023, and OMS721 (79); or the spleen tyrosine kinase inhibitors (80). Currently, precision medicine has not found its place in the treatment of vasculitis (81).

## New Insights in the Follow-up of Patients With IgAV

It is proposed to follow-up patients with IgAV for at least 6–12 months even if the initial blood pressure measurements and urinalysis are normal (74), measuring regularly blood pressure and performing urinalyses to detect presence of haematuria, and quantification of albuminuria and/or proteinuria. Bearing in mind that recently published research has indicated that a certain group of patients (such as older children with the onset of gastrointestinal symptoms before other IgAV symptoms and severe GI form of IgAV, as well as those who develop ulcerations and necroses and persistent purpura) may be at higher risk for the later development of nephritis, the question arises whether some children should be monitored longer than recommended (54, 59).

The question of how long to follow-up children who have developed IgAVN and entered disease remission is still unanswered. During 23 years of follow-up, it was shown that up to 44% of patients with severe IgAVN at onset and up to 13% with mild IgAVN at onset developed reduced renal function and/or hypertension (12). Another study indicated that 70% of pregnancies in women with IgAVN with onset in childhood were complicated by hypertension and/or proteinuria (82). These data call for caution and emphasize the need for long-term observation even in patients who went into remission.

## Conclusion

In this review, we describe how new insights have been gained regarding this disease, which will necessarily require a paradigm shift if we want to make further progress in terms of developing a non-invasive diagnosis of nephritis (which is the most common chronic complication of the disease), as well as its treatment (the main problem being the lack of high-level evidence based on randomized controlled trials). The incidence of IgAV and IgAVN varies worldwide and may be a consequence of overlapping genetic and environmental factors. Besides HLA class II genes, various non-HLA genes may also have significance in its etiopathogenesis. Factors other than Gd-IgA_1_-containing immune complexes may also be important in a multi-hit pathogenesis of this disease. Renal biopsy is still the gold standard for the diagnosis of IgAVN, but in interpretating the histologic findings it should be taken into account that tubulointerstitial changes could be very important as predictors of poor outcome, so other histologic classifications than ISKDC, such as the revised Oxford classification (MEST-C score) may be considered. The most consistent biomarker in patients with IgAV is represented by increased serum levels of Gd-IgA1, while non-invasive confirmation of nephritis is still pending. In the absence of high-level evidence concerning treatment based on randomized controlled trials, SHARE recommendations have been developed. Patients with IgAV, and especially with IgAVN, should be followed-up for long-term, even when the remission of the disease is established, because of possible complications.

## Author Contributions

MJ and MS reviewed the literature and wrote much of the manuscript. TG and RC reviewed the literature and wrote parts of the manuscript, planned and oversaw the entire review, and contributed to all aspects of the manuscript. All authors contributed to the article, approved the submitted version, and agree to be accountable for the content of the work.

## Funding

This work has been supported in part by Croatian Science Foundation under the project IP-2019-04-8822.

## Conflict of Interest

The authors declare that the research was conducted in the absence of any commercial or financial relationships that could be construed as a potential conflict of interest. The reviewer DP declared past co-authorships with one of the authors RC and the absence of any ongoing collaboration with any of the authors to the handling editor.

## Publisher's Note

All claims expressed in this article are solely those of the authors and do not necessarily represent those of their affiliated organizations, or those of the publisher, the editors and the reviewers. Any product that may be evaluated in this article, or claim that may be made by its manufacturer, is not guaranteed or endorsed by the publisher.
